# Surface–Bulk Correlation Spectroscopy for the Characterization of Ternary Adsorption

**DOI:** 10.1177/00037028251350675

**Published:** 2025-06-30

**Authors:** Mahsa Torkamanasadi, Dennis K. Hore

**Affiliations:** 1 Department of Chemistry, 8205 University of Victoria, Victoria, British Columbia, V8W 3V6, Canada; 2 Department of Computer Science, 8205 University of Victoria, Victoria, British Columbia, V8W 3P6, Canada

**Keywords:** Ternary mixtures, adsorption, nonlinear vibrational spectroscopy, heterospectral two-dimensional correlation spectroscopy, 2D-COS ‌

## Abstract

A method is proposed whereby the range of equilibrium constants that describe how a ternary mixture of molecules is adsorbed onto a solid substrate may be obtained. This information may then be related to the surface preference of a particular species. The technique utilizes infrared and/or Raman spectra of the bulk phase, together with sum frequency generation spectra of the surface in a heterospectral two-dimensional correlation analysis. The method consists of a series of up to three experiments in which the bulk concentrations are varied, followed by a set of rules for interpreting the resulting correlation maps to identify adsorption sequences and surface preference. A scheme is provided to direct the design of the experiments and analyses for such multi-component mixtures.

## Graphical abstract

**Graphical abstract:**
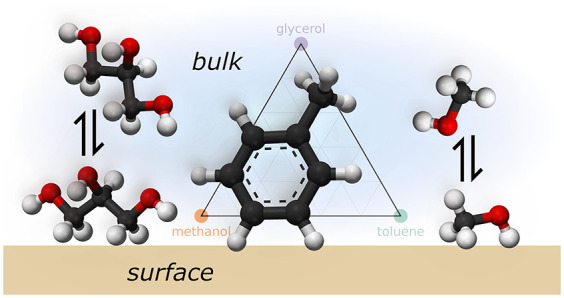
This is a visual representation of the abstract.

## Introduction

Ternary mixtures can exhibit complex phase behavior, surface affinities, and adsorption dynamics. Understanding adsorption behavior in ternary systems is crucial for various industrial, environmental, and scientific applications.^1–4^ These interactions govern the efficiency of chemical separations, catalysis, drug delivery, water treatment, and biofuel production.^5–11^ Surface affinity—an indicator of which component preferentially adsorbs at an interface—affects the stability, composition, and separation efficiency in many of these systems. In biodiesel production, for example, the adsorption of methanol, lipids, and catalyst influences phase separation, reaction efficiency, and product purification.^12–14^ Similarly, in water treatment, polyelectrolyte adsorption onto contaminants impacts flocculation and filtration processes.^15,16^

Recent studies have highlighted the intricate nature of adsorption in ternary systems, especially concerning competitive adsorption behaviors. For instance, the adsorption of heavy metals such as Cu^2+^, Cd^2+^, and Ni^2+^ onto biochar in the presence of microplastics demonstrated that the presence of additional components can significantly influence adsorption capacities and mechanisms.^17,18^ Such findings underscore the importance of understanding multi-component interactions in adsorption processes.

In general, the characterization of surface composition is challenging in cases where the same components are present in an adjacent bulk phase, primarily because most measurement methods would therefore be overwhelmed with signals from the bulk contributions. Spectroscopic methods are particularly valuable due to their ability to probe buried interfaces since they require only access by light. However, spectroscopic characterization of surfaces has an additional challenge in that the markers from surface species may coincide with those in the bulk phase. For this reason, second-order nonlinear optical techniques such as vibrational sum-frequency generation (SFG) spectroscopy are powerful in their ability to provide label-free fingerprints of molecules at surfaces, at sub-monolayer coverage, and inherently exclude contributions from the bulk due to the symmetry requirements of the underlying even-order response function.^19–21^

We have previously illustrated how a combination of infrared (IR) and/or Raman spectra of the bulk phase can be used together with surface SFG spectra to determine preferential adsorption in binary systems.^22–27^ This was based on the application of two-dimensional correlation analysis (2D-COS)^28–31^ that revealed synchronous and asynchronous changes between features (vibrational bands) in the surface and bulk spectra. We now seek to extend this method to study adsorption in ternary mixtures, investigating the role of surface preference in heterogeneous systems. We propose a rule-based approach derived from 2D-COS heterospectral analysis^30,32–35^ that can be used to characterize a particular class of ternary system. By systematically analyzing the interactions and adsorption behaviors within these mixtures, we aim to provide insights that can inform the design and optimization of processes in various applications, from environmental remediation to industrial separations.

## Background and Methods

### Surface Excess Determination from 2D-COS: Comparison with an Approach Developed for Binary Systems

We have previously described a method for determining the surface preference in the case of a binary system using 2D-COS.^22–26^ One of the inherent challenges is that the SFG peak amplitude is related to both the surface population and the orientation/conformation of the molecules at the surface. Furthermore, some species may reorient as they pack closer together. Therefore, before a particular band in the SFG spectra can be used for surface preference determination, one must first verify that the orientation of that particular moiety is not dependent on the surface population. We have previously shown that the way to determine this, without any spectral fitting, is to look at two different vibrational modes associated with the same moiety or that are connected to a rigid part of the molecule.^26^ If there is no SFG homospectral asynchronous cross peak between these frequencies as the bulk concentration changes, then we can be assured that the SFG amplitude is directly proportional to the surface population. Although we previously noted that this then enables the sign of the same cross peak to be analyzed in the surface–bulk (e.g., SFG–IR) heterospectral asynchronous map, we now realize that there was a missed opportunity, as *any* peak could actually be used in the final step, including the surface–bulk “diagonal” peaks. Note that, although it is not customary to see diagonal peaks in asynchronous maps, these occur as we are performing a heterospectral correlation analysis. The relationship between the sign of a heterospectral asynchronous peak and the surface preference of that species simply follows Noda’s rules,^28^ taking the direction of the perturbation into account, namely the direction in which the bulk concentration changes.

In binary systems, the experiment to be performed is obvious, as the bulk mole fractions of the components should be changed, and this becomes the perturbation in the subsequent 2D-COS analysis. However, in the case of the ternary systems we will describe here, the design of the necessary experiments requires some consideration.

### Model Ternary System

Although there are many possibilities for how three components can interact with a surface in a ternary system, we have chosen to study the particular situation where there are three species A, B, and C with a superscript b designating their presence in the bulk solution phase and s when they present on the surface.

(1) $$C^{s}⇌A^{s}⇌B^{s}$$

In this system, C^s^ can be replaced by A^s^ by a process where C desorbs from the surface and becomes C^b^ and a molecule A^b^ adsorbs to become A^s^. A similar exchange can occur for species A and B. These interactions are governed by two equilibrium constants $K_{1}$ and $K_{2}$ that are specified according to

(2\rm a) $$A^{s}+B^{b}+C^{b}\;\overset{K_{1}}{\underset{K_{1}^{-1}}{⇌}}\;A^{b}+B^{s}+C^{b}$$

(2\rm b) $$A^{b}+B^{b}+C^{s}\;\overset{K_{2}}{\underset{K_{2}^{-1}}{⇌}}\;A^{s}+B^{b}+C^{b}$$

implying that

(3\rm a) $$K_{1}=\frac{x_{A}^{b}x_{B}^{s}}{x_{A}^{s}x_{B}^{b}}$$

(3\rm b) $$K_{2}=\frac{x_{A}^{s}x_{C}^{b}}{x_{A}^{b}x_{C}^{s}}$$

where *x* refers to the mole fraction of a particular species in the bulk phase or with respect to the surface coverage. In this system, we are assuming that the surface is fully covered by molecules A, B, or C such that $x_{A}^{s}+x_{B}^{s}+x_{C}^{s}=1$ . We also assume that the population of molecules in the bulk phase is so much larger than the surface population that the bulk mole fractions are not altered when any species adsorbs on the surface. This leads us to expressions for the surface mole fractions

(4\rm a) $$x_{A}^{s}=\frac{K_{2}x_{A}^{b}}{1-x_{A}^{b}-x_{B}^{b}+K_{2}x_{A}^{b}+K_{2}K_{1}x_{B}^{b}}$$

(4\rm b) $$x_{B}^{s}=\frac{K_{1}K_{2}x_{B}^{b}}{1-x_{A}^{b}-x_{B}^{b}+K_{2}x_{A}^{b}+K_{2}K_{1}x_{B}^{b}}$$

(4\rm c) $$x_{C}^{s}=1-x_{A}^{s}-x_{B}^{s}$$

### Infrared, Raman, and Sum-Frequency Generation Spectra

**Figure 1.**
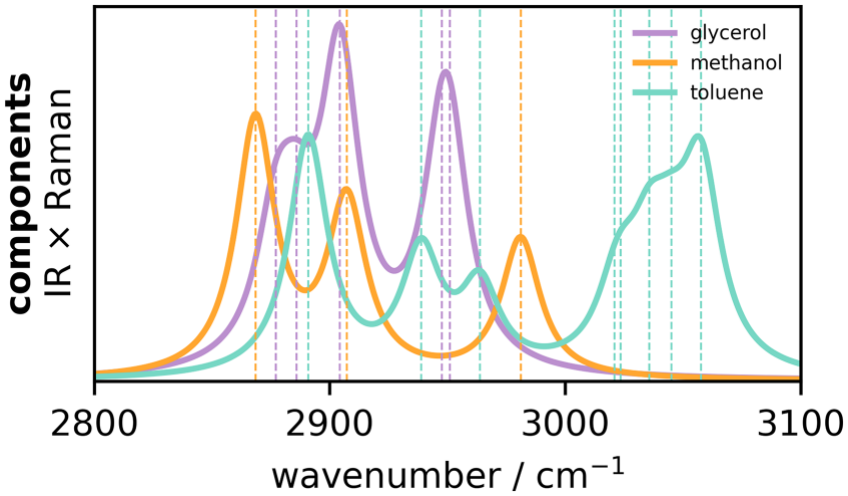
Bulk (IR–Raman hybrid) spectra of the three pure components considered in the ternary mixture. Vertical lines indicate the frequencies of the normal modes, color-matched to the spectra. The same colors will be used to mark frequencies of interest in the subsequent 2D-COS maps.

As an example, we have chosen to work with simulated vibrational spectra consisting of mixtures of glycerol, methanol, and toluene. Ternary mixtures of these molecules are of interest in the purification of glycerol recovered from biodiesel production.^10,11^ Simulated spectra provide a unique opportunity for this proof-of-concept since we can control the equilibrium constants $K_{1}$ and $K_{2}$ and therefore know the relationship between the true surface preference and the rules that we seek to establish from the signs of the 2D-COS peaks. Electronic structure calculations of glycerol, methanol, and toluene were carried out using GAMESS^36^ version 5 at the B3LYP/6-311G(d,p) level. Following a geometry optimization, normal modes were calculated from the Hessian matrix. The IR absorption coefficient was then approximated as the squared derivative of the dipole moment with respect to the normal mode coordinate, evaluated at the equilibrium geometry. Similarly, the Raman scattering cross section was obtained from the squared derivative of the polarizability with respect to the normal mode coordinate. We have previously demonstrated that, although it is possible to use either IR or Raman spectra as a measure of the bulk liquid composition, a hybrid IR–Raman spectrum offers some advantages when used in a heterospectral correlation analysis with SFG spectra.^24^ This is because the SFG oscillator strength is a product of the IR transition dipole moment and Raman transition polarizability, additionally scaled by a factor that takes the orientation distribution into account. If a combined IR–Raman intensity is used for the bulk spectra (based on an isotropic average of each), then the shape of the bulk spectrum more closely matches that of the SFG spectrum and is better suited for a subsequent population analysis in 2D-COS. Details for generating such a hybrid spectrum in the case of simulated and experimental spectra have been described previously.^24,27^ In short, we simply create a Lorentz oscillator with an amplitude that comes from the product of the IR and Raman responses. The IR–Raman hybrid bulk spectra of the three pure components are shown in Figure 1. For the SFG spectra, a similar procedure was applied, except that the amplitude of each vibrational mode comes from the product of the (unsquared) transition dipole moment and transition polarizability, so the spectral bands can be positive or negative. Furthermore, SFG spectra (formally the imaginary component of the second-order susceptibility) need to be computed for a particular orientation distribution. IR and Raman amplitudes are considered an isotropic orientation distribution, but the isotropic SFG response is zero, lending the technique its sought surface-specificity. We therefore model the molecules with a narrow upright distribution, so that their longest axis is perpendicular to the surface.

## Results and Discussion

### General Approach

**Figure 2.**
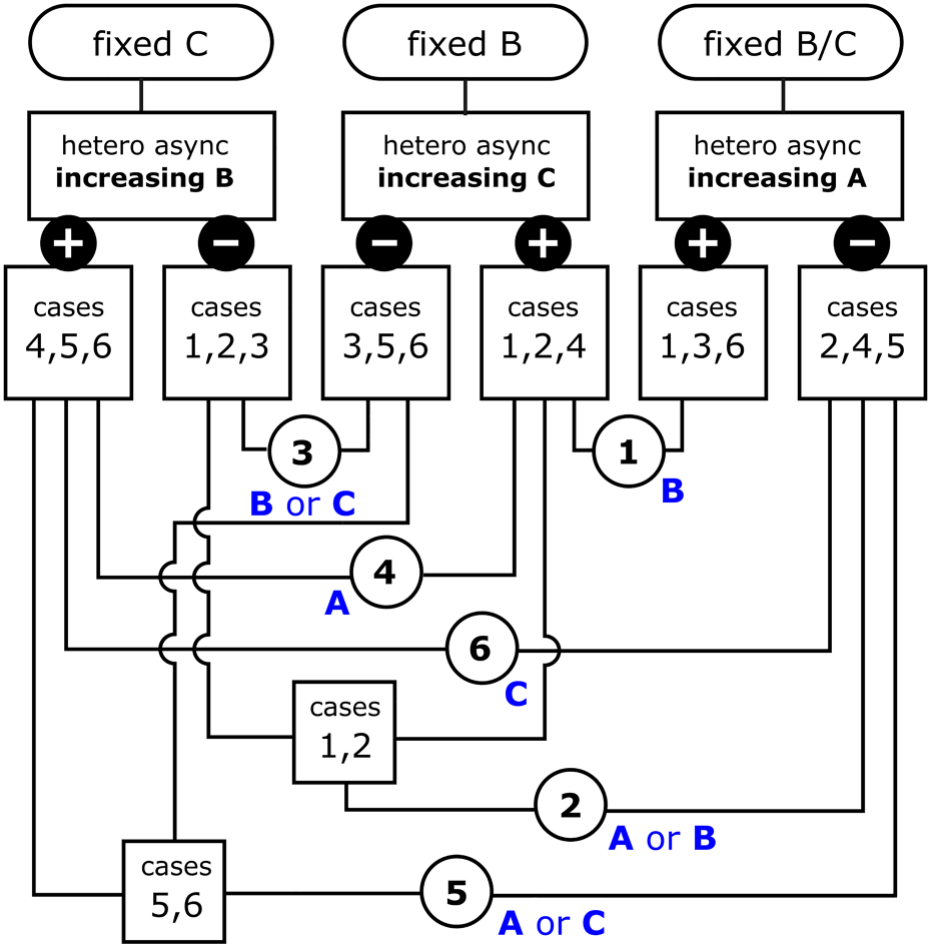
A flowchart illustrating the three potential perturbations required in order to determine which of the six cases (see Table I) characterize the ternary system under investigation. The resulting cases 1–6 must be arrived at using a Boolean AND operation. Only two of the three proposed experiments are required, but those may not be known in advance. Text in blue indicates the surface preference that results from that particular combination of equilibrium constants.

In this particular ternary system, the surface populations are determined by $K_{1}$ , $K_{2}$ , and their ratio $K_{1}/K_{2}$ . There are six possible combinations of these three parameters, that we label as cases 1–6 in Table I. We note that in two instances (cases 3 and 4), the scale of the $K_{1}/K_{2}$ ratio is already known based on the relative magnitude of $K_{1}$ and $K_{2}$ . In some cases (1, 4, and 6) these relative magnitudes are also enough to determine the surface preference as indicated in the fourth column of Table I. As an example, when $K_{1}$ , $K_{2}$ , and their ratio are all greater than unity, Eq. 1–4 can be used to show that the surface prefers species B (case 1). However, in other cases (2, 3, and 5), two possibilities for the preferred adsorbate remain, as the surface preference depends on the specific bulk concentration and the exact values of $K_{1}$ and $K_{2}$ . However, as we seek to use only information based on the signs of the peaks in the heterospectral asynchronous maps, we therefore determine only the relative magnitudes of $K_{1}$ , $K_{2}$ , and their ratio as described by cases 1–6. The task of determining which of the six cases described in Table I characterizes a ternary adsorption system such as that in Eq. 1 requires at least two different experiments (two unique perturbations of bulk mole fractions) and possibly a third one. Since species C and B can be exchanged on the surface only through the intermediate A being adsorbed, one cannot fix the bulk concentration of A in any of the experiments. The three possible experiments are indicated in Figure 2. The first one describes a perturbation where the mole fraction of species C in the bulk $x_{C}^{b}$ is fixed and $x_{A}^{b}$ and $x_{B}^{b}$ are varied. The next experiment is similar, but with $x_{B}^{b}$ fixed. In the final experiment, the ratio $x_{B}^{b}/x_{C}^{b}$ is fixed. If no prior knowledge or chemical intuition can be used to guess information about $K_{1}$ and $K_{2}$ , then one can perform the experiments in any order.

**Table I.** The ternary system shown in Eq. 1 is characterized by two equilibrium constants, $K_{1}$ and $K_{2}$ . Characterization of this system therefore depends on whether $K_{1}$ , $K_{2}$ , and the ratio $K_{1}/K_{2}$ is greater than, less than, or equal to unity. This results in six unique situations illustrated here.

Case	$K_{1}$	$K_{2}$	$K_{1}/K_{2}$	Surface preference	Example
1	> 1	> 1	≥ 1	B	Methanol
2	> 1	> 1	< 1	A or B	Glycerol or methanol
3	> 1	< 1	> 1 by definition	B or C	methanol or toluene
4	< 1	> 1	< 1 by definition	A	glycerol
5	< 1	< 1	< 1	A or C	Glycerol or toluene
6	< 1	< 1	≥ 1	C	Toluene

We therefore start with the two simplest experiments, where $x_{C}^{b}$ is fixed, and the heterospectral 2D-COS are calculated in the direction where $x_{B}^{b}$ increases; and also where $x_{B}^{b}$ is fixed and $x_{C}^{b}$ increases. If the sign-corrected asynchronous peak corresponding to a vibrational mode for species A in the fixed $x_{C}^{b}$ experiment is positive (cases 4, 5, or 6) and a peak for A is also positive in the fixed $x_{B}^{b}$ experiment (cases 1, 2, or 4), the flowchart in Figure 2 indicates that we already know that we are observing a system described by case 4, i.e., $K_{1}<1$ and $K_{2}>1$ . On the other hand, if the corrected asynchronous peak for species A is negative in the fixed $x_{B}^{b}$ experiment (cases 3, 5, or 6), we have eliminated the possibility of cases 3 and 4, but we cannot know whether the set of equilibrium constants correspond to those described in case 5 or case 6. In other words, we know that both $K_{1}$ and $K_{2}$ are smaller than unity, but we have not assessed their ratio. In this situation, a third experiment is required. In the last experiment, in which the ratio $x_{B}^{b}/x_{C}^{b}$ is fixed, a negative peak for species A would indicate $K_{1}/K_{2}<1$ (case 5), while a positive peak reveals that $K_{1}/K_{2}\geq 1$ (case 6). We will now provide a detailed example that illustrates the use of these rules using model vibrational spectra.

### Example

We consider a ternary mixture where species A is glycerol, species B is methanol, and species C is toluene. For the sake of this illustration, we have generated model spectra using $K_{1}=5$ and $K_{2}=1.5$ . Following the procedure we have outlined above, we need to perform at least two perturbations. However, as we aim to determine which of the six cases (secretly knowing that we have modeled case 1) in Table I describes our system based on the signs of the heterospectral asynchronous cross peaks alone, a third perturbation may be required.

As we have mentioned, one normally needs to ensure that surface species do not reorient as their surface population changes, prior to the interpretation of the heterospectral correlation peaks. However, as this is a simulation, we have simplified the situation by ensuring that the surface orientation remains constant. We therefore do not need to examine the surface (SFG) homospectral correlation, as we know (and have verified) that there are no asynchronous cross peaks; all SFG signals can therefore readily be interpreted in terms of surface population changes.

## Perturbation 1: Fixed Bulk Methanol Concentration

**Figure 3.**
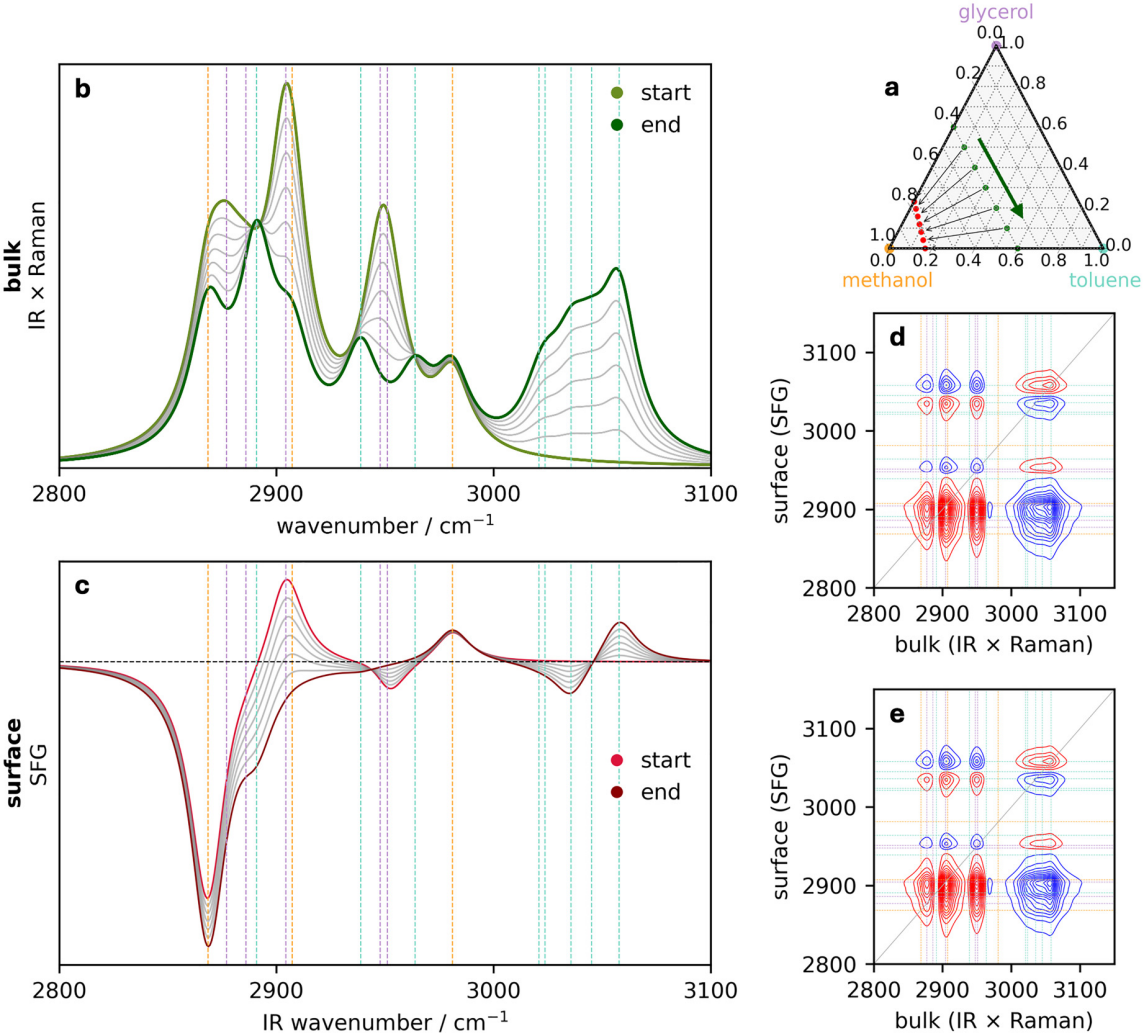
Perturbation 1: The bulk methanol mole fraction remains fixed. (a) Ternary diagram illustrating the initial bulk concentrations of the three components (green circles) that define the perturbation along the direction indicated by the arrow. The surface concentrations are provided by Eq. 4a and 4b and are indicated by the red circles. The corresponding series of (b) bulk (IR–Raman hybrid) and (c) surface (SFG) spectra. Surface–bulk heterospectral correlation analysis showing the (d) synchronous and (e) asynchronous maps. In all spectra, the vertical dashed lines are colored corresponding to the assignment of the pure spectral components in Figure 1.

We first investigate the perturbation in which the methanol mole fraction is fixed at 0.4. Seven mixtures were considered, with varying bulk concentrations of glycerol and toluene comprising the remaining 60% of the molecules. Those bulk compositions are shown with green circles in Figure 3a. The direction of the perturbation, increasing bulk toluene, is indicated by the arrow in Figure 3a, and the corresponding bulk (IR–Raman) spectra are shown in Figure 3b. Since we know the *K* values, we are able to calculate the surface concentrations from Eq. 4; those are indicated with the red circles in Figure 3a. In a realistic scenario, those points would not be known, and we would have an indication of those surface compositions only through the observation of the SFG spectra shown in Figure 3c.

For both bulk and surface spectra, vertical lines mark the wavenumbers of characteristic bands originating from the individual molecules, using colors corresponding to those shown in Figure 1. We now turn to the heterospectral 2D-COS analysis presented in Figures 3d and 3e, and first note that the unusual situation where the synchronous and asynchronous maps identical shapes for all bands. (In this case, the signs of the synchronous and asynchronous peaks are the same too, but we will later see an example where the signs are all opposite to each other.) This situation occurs because, in the absence of molecular reorientation, the SFG and bulk IR–Raman spectra are just scaled according to the corresponding surface and bulk populations. This is especially evident in our simulated spectra as there is no noise but in practice, the same would be observed experimentally if none of the species reoriented. We will come back to the point about surface reorientation later. But first, we seek to identify glycerol or toluene bands in relatively uncongested spectral regions where we can be more sure of the band assignment. In this case, it is straightforward to look at toluene aromatic C–H stretching, as those signals uniquely appear above 3000 cm^−1^. In the absence of surface reorientation, we can study any asynchronous toluene peak in Figure 3e, including diagonal peaks. We notice that the diagonal peak near 3040 cm^−1^ is negative (blue), and the corresponding synchronous peak is also negative. Note that the 3060 cm^−1^ peak is positive in both maps and has a different sign from the 3040 cm^−1^ feature due to the difference in sign in the SFG response. Either of those toluene modes can be used for this analysis, where Noda’s rules^28^ indicate that the bulk toluene concentration increases before the surface toluene concentration increases. The surface avoids toluene, characterized by cases 1, 2, and 4. In order to narrow down the possibilities among these three cases, at least one additional perturbation is required.

## Perturbation 2: Fixed Bulk Toluene Concentration

**Figure 4.**
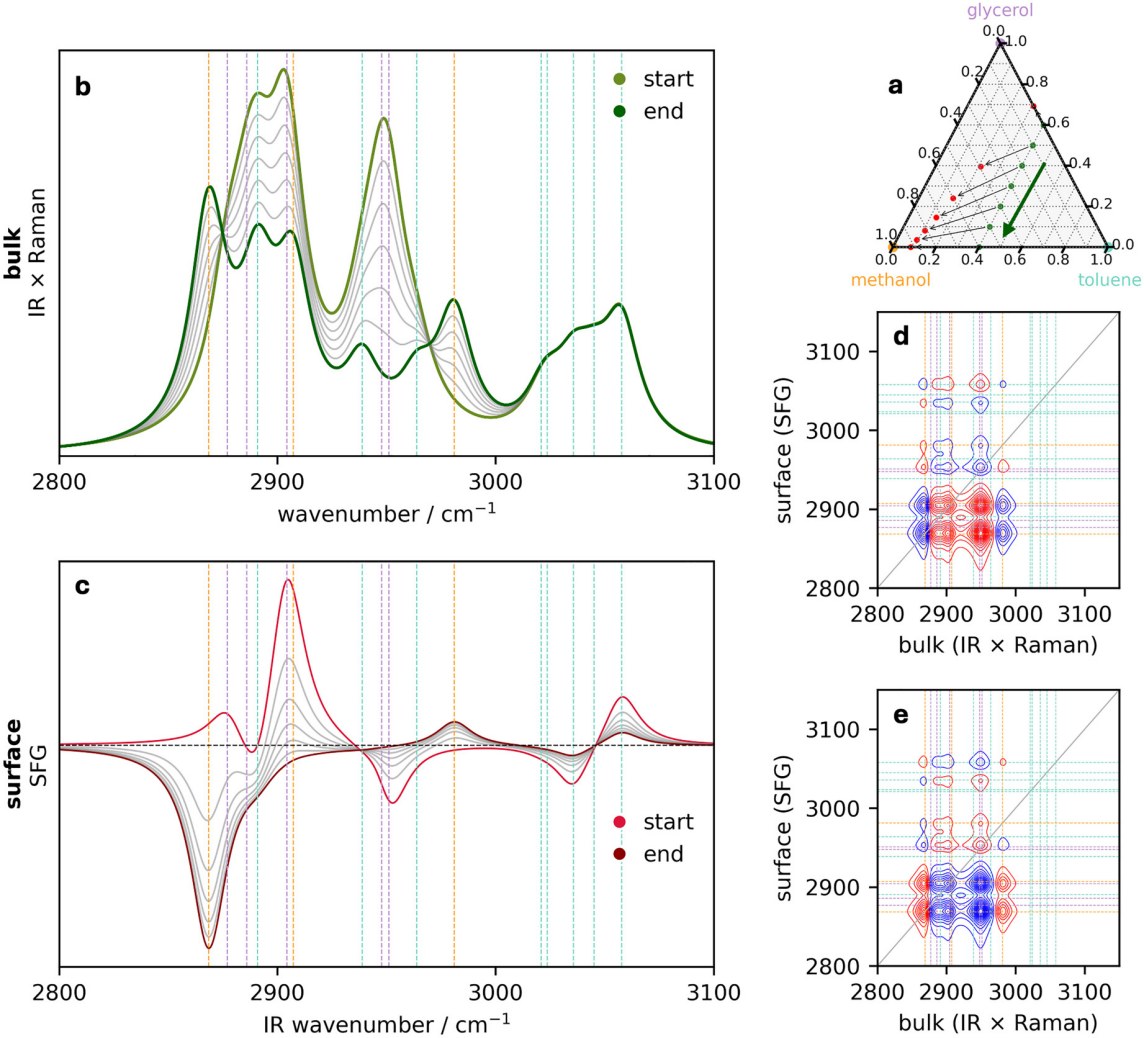
Perturbation 2: The bulk toluene mole fraction remains fixed. (a) Ternary diagram illustrating the initial bulk concentrations of the three components (green circles) that define the perturbation along the direction indicated by the arrow. The surface concentrations are provided by Eq. 4a and 4b and are indicated by the red circles. The corresponding series of (b) bulk (IR–Raman hybrid) and (c) surface (SFG) spectra. Surface–bulk heterospectral correlation analysis showing the (d) synchronous and (e) asynchronous maps. In all spectra, the vertical dashed lines are colored corresponding to the assignment of the pure spectral components in Figure 1.

The next experiment that is performed keeps the bulk toluene concentration fixed at 0.4 mole fraction while the bulk methanol fraction is increased as indicated by the green circles in Figure 4a. The corresponding bulk spectra are shown in Figure 4b, and the surface spectra in Figure 4c. We are now looking for either glycerol or methanol bands in the 2D-COS maps, noting the absence of any toluene peaks. This is more challenging as the aliphatic C–H stretching region is relatively crowded. However, we have managed to identify a methanol diagonal peak at 2870 cm^−1^ corresponding to the methyl symmetric stretch. This peak is positive (red) in the asynchronous map (Figure 4e) and negative (blue) in the synchronous map (Figure 4d) indicating that the surface methanol population increases before the bulk population increases. We therefore know that the surface prefers methanol. Looking at the flowchart in Figure 2, this result corresponds to cases 1, 2, or 3. If we combine this information from what we learned from the fixed methanol experiment, we see that the subset of these possibilities leaves cases 1 or 2, indicating that both $K_{1}$ and $K_{2}$ are greater than unity. Distinguishing these remaining two cases amounts to resolving the ratio $K_{1}/K_{2}$ , and requires a third experiment.

## Perturbation 3: Fixed Bulk Methanol/Toluene Ratio

**Figure 5.**
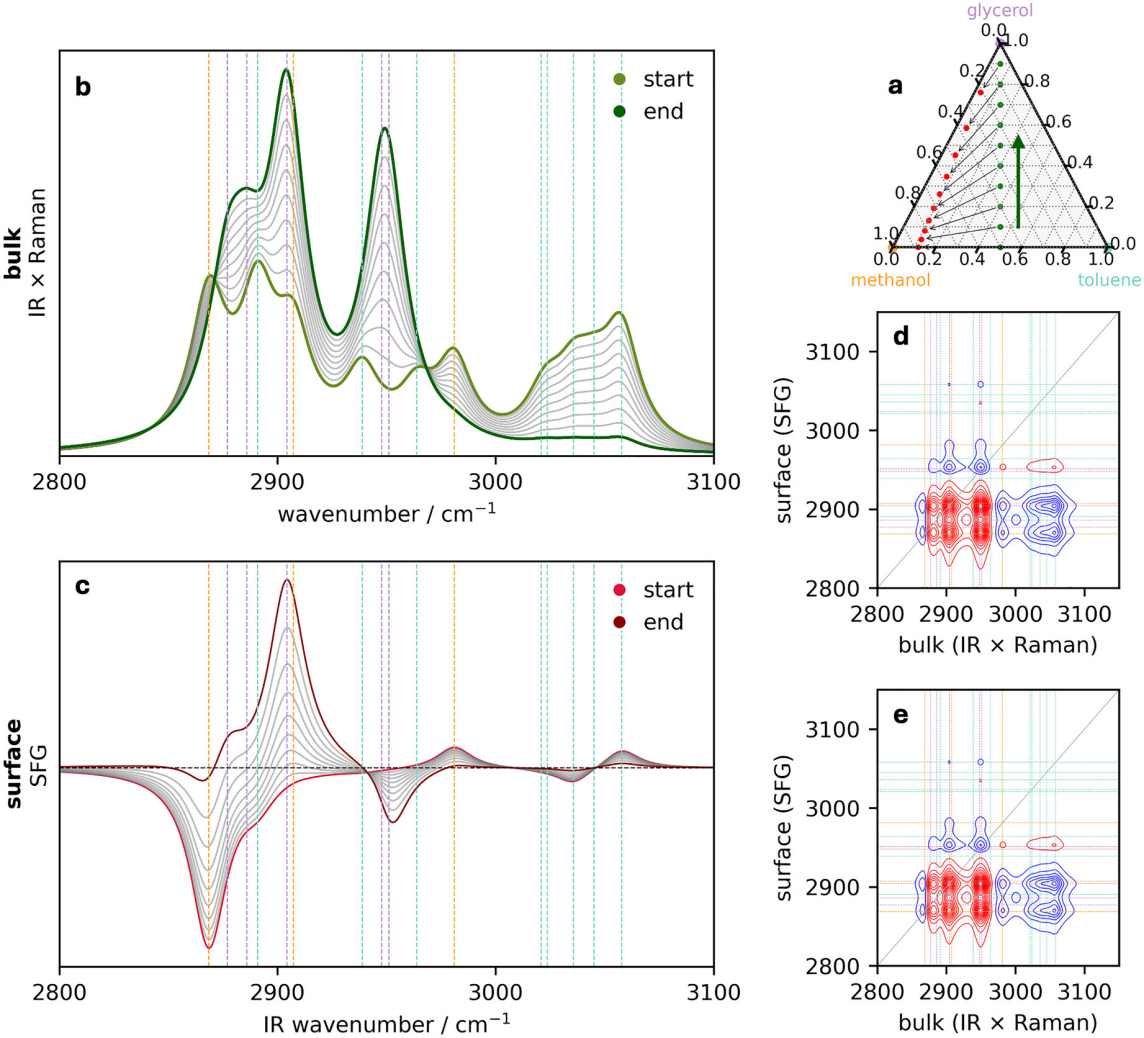
Perturbation 3: Methanol/toluene ratio remains fixed. (a) Ternary diagram illustrating the initial bulk concentrations of the three components (green circles) that define the perturbation along the direction indicated by the arrow. The surface concentrations are provided by Eqs. 4a and 4b and are indicated by the red circles. The corresponding series of (b) bulk (IR–Raman hybrid) and (c) surface (SFG) spectra. Surface–bulk heterospectral correlation analysis showing the (d) synchronous and (e) asynchronous maps. In all spectra, the vertical dashed lines are colored corresponding to the assignment of the pure spectral components in Figure 1.

In the final perturbation, the concentrations of methanol and toluene are decreased in the bulk while keeping their ratio fixed at equal proportions, as indicated by the green circles in Figure 5a. The corresponding bulk spectra are shown in Figure 5b, and surface spectra in Figure 5c. We now inspect the peaks which appear in the asynchronous 2D-COS map. There is no diagonal peak at 3040 cm^−1^, which reveals that as the bulk toluene mole fraction is varied, the surface mole fraction of toluene remains small, suggesting that the surface avoids toluene. As we wish to use information based on the signs of the asynchronous peaks, we examined the diagonal mode corresponding to the methyl symmetric stretch of methanol. In the 1D spectra, this appears at 2870 cm^−1^. However, the center of the band in the 2D-COS is shifted to slightly lower frequencies due to interference. The corrected sign here is positive, indicating that the bulk change happens before the surface change. As our chosen perturbation decreased methanol in the bulk, this means the decrease in the bulk occurs before the decrease at the surface. This leads us to understand that the surface favors methanol. By referring back to Figure 2, we now know that the ratio $K_{1}/K_{2}\geq 1$ . Among the possible scenarios of cases 1, 3, and 6 in general where $K_{1}/K_{2}>1$ , when we consider this result together with what we have learned from the previous two experiments, the only consistent possibility is case 1. Putting everything together, since $K_{1}$ , $K_{2}$ , and the ratio $K_{1}/K_{2}$ are all greater than unity, we can conclude that this surface consistently prefers methanol.

### Additional Considerations

Throughout the discussion, we have lumped together cases where the equilibrium constants or their ratio are greater/less than or equal to unity, but we have not described the situations where $K_{1}$ or $K_{2}\approx 1$ or $K_{1}/K_{2}\approx 1$ in detail. In general, when either of the equilibrium constants is close to unity, there is no strong preference for adsorption, and the surface composition of a particular species is roughly the same as the bulk composition. In the 2D-COS analysis we have presented, this is revealed by the absence of asynchronous peaks for that species. However, the situation where $K_{1}$ and $K_{2}$ are both significantly larger or smaller than unity and $K_{1}\approx K_{2}$ , i.e., $K_{1}/K_{2}\approx 1$ deserves some special consideration. By inspecting Eq. 4, we can see that if $K_{1}\approx K_{2}>1$ then we are in case 1, where the surface prefers species B. On the other hand, if $K_{1}\approx K_{2}<1$ the surface prefers species C (case 6).

One of the primary motivations of this work has been to understand the surface preference for selectively adsorbing specific molecules from a bulk liquid. In the case of a binary system, such a conclusion directly follows from the application of the rules we have discussed for interpreting heterospectral surface–bulk 2D-COS maps. However, there are many possibilities for how species are adsorbed/desorbed and interconverted in ternary systems. In our chosen system (Eq. 1), the characterization of the set of equilibrium constants resulted in six possibilities, and we have demonstrated how a judicious choice of up to three perturbations can be used to isolate one of the six combinations. However, some of those combinations do not yield conclusive information on the surface preference. Looking at Table I, we can see that the surface preference is determined for cases 1, 4, and 6. In other words, in those three combinations of $K_{1}$ , $K_{2}$ , and the ratio $K_{1}/K_{2}$ , the surface preference is independent of the exact value $K_{1}$ and $K_{2}$ , as was in the case in the example we presented. However, in cases 2, 3, and 5, more detailed knowledge of $K_{1}$ and $K_{2}$ and the mole fractions in the bulk are required in order to make definitive conclusions about the surface preference, which needs to then come directly from Eq. 4.

Another point in general concerns the reorientation of species when their surface population changes. This is relatively common, for example, as the packing density of adsorbed monolayers often moves their tilt angle to more upright orientations. In our earlier description of binary systems,^26^ we noted that if one of the two species reoriented on the surface (as evidenced by SFG homospectral asynchronous features between two vibrational modes corresponding to the same rigid moiety), then it may still be possible to have a full surface preference determination based on the other species. In the case of ternary systems, this is still true. If one component does not reorient in each experiment, it may still be possible to navigate the flowchart in Figure 2 and arrive at a conclusion. Further details of this situation may be investigated in future work.

## Conclusion

We have provided a framework where IR and/or Raman spectra can be used together with vibrational SFG spectra to characterize a ternary system with the aid of heterospectral 2D-COS. The primary merit of this approach is that the direction of the equilibria can be determined solely from the signs of the heterospectral asynchronous peaks. This work therefore provides an initial step toward the understanding of more complex adsorption phenomena and adsorption dynamics in ternary systems. This preliminary study provides a scientific foundation for optimizing material design, improving industrial separation techniques, and understanding reaction kinetics in complex mixtures.
